# Emotional reactivity to daily life stress in spousal caregivers of people with dementia: An experience sampling study

**DOI:** 10.1371/journal.pone.0194118

**Published:** 2018-04-04

**Authors:** Rosalia J. M. van Knippenberg, Marjolein E. de Vugt, Rudolf W. Ponds, Frans R. J. Verhey, Inez Myin-Germeys

**Affiliations:** 1 Department of Psychiatry and Neuropsychology and Alzheimer Center Limburg, School for Mental Health and Neurosciences, Maastricht University, Maastricht, the Netherlands; 2 Department of Neurosciences, Center for Contextual Psychiatry, KU Leuven, Leuven, Belgium; National University of Ireland Maynooth, IRELAND

## Abstract

**Introduction:**

Caregivers differ in their emotional response when facing difficult situations during the caregiving process. Individual differences in vulnerabilities and resources could play an exacerbating or buffering role in caregivers’ reactivity to daily life stress. This study examines which caregiver characteristics modify emotional stress reactivity in dementia caregivers.

**Methods:**

Thirty caregivers collected momentary data, as based on the experience sampling methodology, to assess (1) appraised subjective stress related to events and minor disturbances in daily life, and (2) emotional reactivity to these daily life stressors, conceptualized as changes in negative affect. Caregiver characteristics (i.e. vulnerabilities and resources) were administered retrospectively.

**Results:**

Caregivers who more frequently used the coping strategies ‘seeking distraction’, ‘seeking social support’, and ‘fostering reassuring thoughts’ experienced less emotional reactivity towards stressful daily events. A higher educational level and a higher sense of competence and mastery lowered emotional reactivity towards minor disturbances in daily life. No effects were found for age, gender, and hours of care and contact with the person with dementia.

**Discussion:**

Caregiver resources can impact emotional reactivity to daily life stress. Interventions aimed at empowerment of caregiver resources, such as sense of competence, mastery, and coping, could help to reduce stress reactivity in dementia caregivers.

## Introduction

Worldwide, 47 million people live with dementia, and this number is expected to increase to more than 131 million by 2050 [1]. The majority of the people with dementia are living at home and are primarily cared for by their spouse or other relatives. Providing years of extensive care for a person with dementia (PwD) is associated with increased levels of stress and a higher risk of developing depression and other adverse health problems [2, 3]. Health problems in the caregiver are often overlooked and overshadowed by those of the PwD. However, caregiver well-being is important for both the caregiver and PwD, since it predicts the quality of care and timing of nursing home placement [4, 5].

Caregivers differ in their emotional response when facing difficult situations during the caregiving process. Several conceptual models [6, 7] have been developed in the past decades to improve our understanding of the relationship between stress, coping, and health. According to a meta-analysis by Vitaliano et al. [8] on stress and physical health in caregivers of people with dementia, previous models share the idea that individual differences, such as vulnerabilities and resources, moderate relationships of stressors with distress. Vulnerabilities and resources can be distinguished according to their stability and changeability among pathways from stressors to illness. The term vulnerability refers to stable, hard-wired characteristics [9], such as age, gender [6], and race [10]. In contrast, resources are more dynamic and mutable characteristics that are affected by interactions with the environment, such as coping and social support [11, 12].

Previous studies [13–17] have indicated several factors to play a role in experienced levels of caregiver distress. A recent systematic review reported that women and older caregivers generally have a higher risk of experiencing stress [13]. In addition, highly educated caregivers more often use effective caregiver management strategies, which suggests that these caregivers are better able to adapt to the care demands [4]. Besides these hard-wired characteristics (i.e. vulnerabilities), dynamic characteristics (i.e. resources) like caregivers’ sense of competence, mastery, and coping strategies have been found to be important indicators of emotional distress [14, 15]. Also, dynamic characteristics of the care recipient can affect caregiver outcomes. The occurrence of problem behavior in the PwD can be unpredictable and is likely to fluctuate on a day-to-day basis [16]. Caregiver distress has been demonstrated to increase when neuropsychiatric symptoms are present and when dementia severity increases [17]. Due to an increase in care intensity caregivers’ quality of life may be negatively impacted [14].

Most studies [18] have used standard retrospective measures to examine determinants and levels of caregiver distress. Retrospective questionnaires are inadequate to capture moment-to-moment fluctuations in stress and rather take a snapshot of daily life. That is, data are mostly collected at only one occasion, and therefore only provide a global view of caregiver distress. Moreover, retrospective measures are highly susceptible to recall biases, which leads caregivers to over- or underestimate stress symptoms [19, 20]. So far, very little research has been conducted to investigate caregiver well-being in-the-moment and in a natural setting. The experience sampling methodology (ESM), also known as ecological momentary assessment [21] or ambulatory assessment [22], is a structured diary method that has been developed over the past decades and can be used to self-monitor subjective experiences in the flow of daily life [23, 24]. The main advantages of the ESM are that it assesses experiences in-the-moment, resulting in less memory biases compared to traditional retrospective measures and it allows for exploration of temporal relationships between variables and revelation of detailed information on daily fluctuations in subjective experiences [25]. This is of particular importance in spousal caregivers of PwD, as they often provide 24/7 care and their stress experiences are likely to fluctuate constantly during the day in response to the ebb and flow of dynamic care-related stressors [16].

The ESM has been applied in several studies on emotional reactivity to daily life stress in psychiatric populations [26, 27]. Moreover, the ESM has been employed before in clinically burned-out patients that are, similar to caregivers, likely to experience prolonged elevated levels of stress [28]. The ESM could provide valuable information on caregivers’ sensitivity to daily life stress, i.e. their emotional responses to daily stressors, and on factors that buffer or exacerbate reactivity to those stressors. It has been demonstrated in the general population that small daily events are important predictors of psychological symptoms and subjective distress [29]. Caregivers’ emotional reactivity to daily life stress might constitute part of the underlying vulnerability for becoming overburdened in a later stage of the caring process. More insight into factors that influence caregivers’ emotional reaction to day-to-day problems could help to identify the relevant elements to focus on in caregiver interventions [30].

### Aims of the study

The current study aims to examine which specific caregiver characteristics, including vulnerabilities and resources, modify emotional reactivity to daily life stress in caregivers of people with dementia.

## Materials and methods

### Participants

Thirty-one informal caregivers entered the study between February 2013 and February 2014. One caregiver was excluded from the analyses for completing fewer than 20 valid ESM reports (less than 33% of the total 60 ESM reports). Caregivers were recruited in the Memory Clinic of the Maastricht University Medical Center Plus (MUMC+), the Atrium Medical Center Parkstad, and in mental health care institutions in the Southern Netherlands. Participants had to meet the following inclusion criteria: (1) being a spousal caregiver of a person diagnosed with dementia; (2) sharing a household with the PwD; and (3) informed consent obtained. Exclusion criteria were: (1) having insufficient cognitive abilities to engage in the ESM; and (2) being overburdened or having severe health problems, as judged on the basis of clinical experience and expertise by a knowledgeable health care professional that is actively involved in the treatment of the PwD.

The Medical Ethical Committee of the MUMC+ (#12-3-049) approved this study. Informed consent was obtained from all caregivers who participated in the study. No informed consent was obtained from the care recipients, as data on the care recipients were collected by proxy and analyzed anonymously to describe the study sample at baseline.

### Study design and procedure

This study concerns an exploratory study with a cross-sectional design. The study protocol for each participant included:

(1) Introductory session: a demographic interview was conducted to assess caregiver vulnerabilities, including age, gender, and education, and care recipient characteristics, including age, gender, education, type of dementia, and dementia severity and duration. Subsequently, participants received an electronic ESM device, the ‘PsyMate’ [31], to collect data in their daily lives. The feasibility of the ‘PsyMate’ in caregivers of PwD has recently been demonstrated [32]. A 30-minute training session was provided to explain the ESM procedure and how to operate the ‘PsyMate’. All participants were provided with a coded ID number that was linked to their PsyMate in order to maintain participant confidentiality.

(2) ESM data collection: participants were asked to collect ESM data with the ‘PsyMate’ for 6 consecutive days, starting the day after the introductory session. The ‘PsyMate’ generated an alert (beep) at ten unpredictable moments per day between 7:30 AM and 10:30 PM. After every alert, participants were asked to immediately complete a questionnaire presented on the screen of the ‘PsyMate’ concerning their current context (location, activity, social company), appraisals of the situation, and negative affect.

(3) Debriefing session: after the ESM data collection participants were asked to complete retrospective questionnaires concerning their resources, including sense of competence, mastery, and coping strategies. In addition, the presence of neuropsychiatric symptoms in the PwD during the past week was assessed.

### Assessment of emotional reactivity to daily life stress

Based on previous ESM studies, emotional reactivity to daily life stress was conceptualized as negative affect reactivity to daily events (event-related stress) and minor disturbances that continually occur in the flow of daily life (activity-related stress) [27, 33]. Negative affect and stress measures were derived from ESM reports as described below. Standardized sets of ESM items are not yet available [34]. Therefore, the choice of the items was made on the basis of information available from previous ESM studies [27, 35], guidelines from ESM experts for designing an ESM study [36], and knowledge about the range of experiences that spousal caregivers of people with dementia could be expected to encounter.

### Assessment of negative affect

Caregivers’ negative affect state reported after each beep was assessed with eight ESM items. The negative affect scale included the items “insecure”, “lonely”, “anxious”, “irritated”, “down”, “desperate”, and “tensed” (Cronbach’s α = .81). The item “confident” had a low loading on the negative affect scale and was excluded.

### Assessment of stress

Two different stress measures were computed:

(1) Event-related stress: after each beep participants were asked to think about the most important event that happened between the current and the previous ESM report. This event could be either positive or negative, such as a pleasant phone call from a friend or a difficult situation with the PwD. Subsequently, participants had to rate on a 7-point bipolar Likert scale (-3 = very unpleasant, 0 = neutral, 3 = very pleasant) whether the event was perceived as pleasant. The negatively or neutrally (-3 to 0) rated events were used to create an event-related stress score that reflects caregivers’ feelings of stress caused by daily events. Item scores were reversed, so that higher mean scores indicated higher levels of event-related stress.

(2) Activity-related stress: after each beep participants had to judge their current activity (e.g. care task, household, relaxation) on four ESM items rated on a 7-point Likert scale (1 = not at all to 7 = very). The mean of the items “I can do this well”, “I like doing this”, “I would rather do something else”, and “this is difficult for me” formed the activity-related stress score (Cronbach’s α = .57). The first two item scores were reversed, so that higher mean scores indicated higher levels of activity-related stress. Compared to the event-related stress score, the activity-related stress score reflects momentary feelings of stress caused by minor disturbances that continually occur in the flow of daily life.

### Caregiver characteristics assessment

#### Assessment of demographics

Information regarding age, gender, and education level was obtained during a demographic interview with the caregiver.

#### Assessment of sense of competence

The Short Sense of Competence Questionnaire (SSCQ) was used to assess the caregiver’s sense of competence. The SSCQ assesses caregiver’s feelings of being capable to care for the PwD and contains seven items rated on a 5-point scale from 1 (agree very strongly) to 5 (disagree very strongly). All items were accumulated into a total SSCQ score (range 7–35). Higher scores indicate more sense of competence. Cronbach’s alfa showed the SSCQ to reach high reliability, α = .88. According to previous research, the SSCQ displays good content and construct validity [37].

#### Assessment of mastery

The Pearlin Mastery Scale (PMS) was used to assess the extent to which a caregiver perceives him- or herself to be in control of events and on-going situations, also known as mastery [38]. The scale contains seven items with scores varying from 0 (complete agree) to 4 (completely disagree). Items were summed to form a total mastery score (range 0–28), with higher scores reflecting greater perceived control. Reliability of the PMS was high (Cronbach’s α = .80).

#### Assessment of coping strategies

The 47-item Utrecht Coping List (UCL) was used to measure seven coping strategies in the caregiver, including ‘seeking distraction’ (8 items, Cronbach’s α = .60), ‘expressing emotions’ (3 items, Cronbach’s α = .54), ‘seeking social support’ (6 items, Cronbach’s α = .88), ‘avoiding’ (8 items, Cronbach’s α = .68), ‘fostering reassuring thoughts’ (5 items, Cronbach’s α = .74), ‘passive coping’ (7 item, Cronbach’s α = .79), and ‘active coping’ (7 items, Cronbach’s α = .92) [39]. Items were rated on a 4-point scale, ranging from 1 (rarely or never use this strategy) to 4 (very often use this strategy). The reliability and validity have been found to be sufficient despite some inconsistencies in the literature [39].

#### Assessment of care intensity

Information regarding weekly hours of contact with and weekly hours of care for the PwD was obtained during a demographic interview with the caregiver.

### Care recipient characteristics assessment

#### Assessment of neuropsychiatric symptoms

The Neuropsychiatric Inventory (NPI) was used to evaluate twelve neuropsychiatric symptoms in the PwD [40]. If a symptom is present, the caregiver rates its frequency and severity on a scale from respectively 1 (rarely) to 4 (very often), and 1 (mild) to 3 (severe). The score for each domain was computed by multiplying the frequency and severity score. Subsequently, a total NPI score was calculated by adding the domain scores together. The Dutch version of the NPI has been found to be an objective and valid rating scale for measuring behavioral and psychological symptoms in dementia [41].

#### Assessment of dementia severity and duration

The Clinical Dementia Rating scale (CDR) was used to stage the severity of dementia in the PwD [42]. The CDR has become widely accepted in the clinical setting as a reliable and valid global assessment measure of dementia [43]. The researcher rated the CDR score on a 5-point scale (0 = “normal”; 0.5 = “very mild dementia”; 1 = “mild dementia”; 2 = “moderate dementia”; and 3 = “severe dementia”) according to information obtained from the caregiver. Reliability of the CDR was high (Cronbach’s α = .93). Additionally, the year of dementia onset was administered.

### Statistical analysis

Participants with fewer than twenty valid ESM reports (less than 33% of the in total 60 ESM reports) were excluded from the analyses [44]. Multilevel modeling techniques were used to account for the hierarchical structure of ESM data, in which multiple observations (beep level 1) are nested within days (day level 2) and days are nested within individuals (individual level 3) [45]. Data were analyzed with the XTMIXED module in STATA 12.1 (StataCorp, College Station, TX). Analyses were conducted separately for the two stress measures (event- and activity-related stress). Negative affect was entered as the dependent variable. Ratings of stress (event- or activity-related stress), caregiver characteristics (age, gender, education level, weekly hours of contact with and care for the PwD, sense of competence, mastery, coping strategies), and their interactions were entered as the independent variables, leading to the following model: negative affect = β0 + β1 stress + β2 caregiver characteristic + β3 (stress x caregiver characteristic) + residual. In addition, care recipient characteristics (i.e. disease severity and duration, and neuropsychiatric symptoms) were included as possible confounders. Post-hoc analyses were performed to test the overall effect of the categorical variables (gender and education level) and to separately test the effect of each categorical level. The interaction term was of most interest in the present study as the main question concerned which caregiver characteristics modify emotional stress reactivity. Stratified analyses were conducted in case of significant interaction effects to examine the direction of the effect in more detail. To this end, participants were classified into tertiles (low, middle, high) according to their score on the concerning caregiver characteristic. For each caregiver characteristic, emotional reaction to daily life stress was analyzed in the three groups separately according to the following model: negative affect = β0 + β1 stress + residual. Graphs were generated to illustrate the data in more detail.

## Results

### Participants and descriptive statistics

Participants completed on average 49.3 out of 60 valid reports (*SD* = 5.2). Table 1 contains the demographic and clinical characteristics of the 30 participating caregivers and their care recipients. Of the caregivers, 60.0% were women (18/30), and 73.3% took care for a spouse diagnosed with Alzheimer’s disease (22/30). Of the care recipients, 73.4% showed a mild severity of dementia (CDR 0.5 or 1: 22/31).

**Table 1:** Demographic and clinical characteristics of the caregivers and care recipients.

Variable	Caregivers (N = 30)	Care recipients (N = 30)
Age (*M*, *SD*, *range*)	69.9 ± 5.8 (57–80)	73.7 ± 6.2 (61–87)
Gender (*n*, %)		
Male	12 (40.0)	18 (60.0)
Female	18 (60.0)	12 (40.0)
Level of education: 1–8 (*n*, %)^a^		
Low	13 (43.3)	16 (53.4)
Middle	8 (26.7)	6 (20.0)
High	9 (30.0)	8 (26.6)
Type of dementia (*n*, %)		
Alzheimer’s disease		22 (73.3)
Vascular dementia		3 (10.0)
Frontotemporal dementia		2 (6.7)
Dementia with Lewy Bodies		1 (3.3)
Mixed dementia		2 (6.7)
Dementia severity–CDR^b^ (*n*, %)		
0.5: very mild		11 (36.7)
1: mild		11 (36.7)
2: moderate		7 (23.3)
3: severe		1 (3.3)
Dementia duration in years (*M*, *SD*, *range*)		6 ± 3.8 (1–15)
Neuropsychiatric symptoms–NPI^c^ (*M*, *SD*, *range*)		13.9 ± 14.3 (1–57)

^a^Educational level was compressed to three levels: low (primary education, including lower vocational), middle (secondary education, including intermediate vocational), and high (higher education, including higher vocational and bachelor’s, graduate, and doctoral degree).

^b^CDR = Clinical Dementia Rating scale

^c^NPI–Neuropsychiatric Inventory

Mean scores on caregivers’ negative affect, both stress measures, the remaining caregiver characteristics as well as its correlations are shown in Table 2. The average ratings of negative affect, event-related stress, and activity-related stress were 1.9 (SD = 0.8), 0.7 (SD = 0.5), and 2.7 (SD = 0.7), respectively. The two stress measures were weakly correlated (*r* = 0.20, *p* correlated (*r* = 0.20, *p* <.001).

**Table 2:** Ratings and correlations of negative affect, stress, and caregiver characteristics (N = 30).

	Score			Correlation (r)^a^										
Measure	Mean	SD	Range	Item 1	2	3	4	5	6	7	8	9	10	11
Negative affect^b^	1.9	0.8	1.0 to 4.4											
Stress measures														
1. Event-related stress^b^	0.7	0.5	0 to 1.8	1.00										
2. Activity-related stress^b^	2.7	0.7	1.5 to 3.9	0.20***	1.00									
Caregiver characteristics														
1. Hours of contact with PwD per week^c^	153.3	12.4	126 to 168	1.00										
2. Hours of care for PwD per week^c^	52.0	55.9	0 to 168	.25***	1.00									
3. Sense of competence (SSCQ)	25.7	6.5	8 to 35	-.16***	-.31***	1.00								
4. Mastery (PMS)	17.4	4.9	5 to 26	-.16***	-.54***	-.59***	1.00							
Coping strategies:														
5. Active coping	20.2	4.3	13 to 28	.37***	-.18***	.32***	.61***	1.00						
6. Passive coping	10.0	2.8	7 to 18	-.19***	.34***	-.41***	-.74***	-.59***	1.00					
7. Seeking distraction	16.9	3.0	11 to 23	-.05	-.27***	.21***	.17***	-.12**	.03	1.00				
8. Expressing emotions	5.5	1.0	3 to 7	-.10*	-.02	-.25***	-.33***	-.27***	.26***	.12***	1.00			
9. Seeking social support	13.0	3.3	8 to 23	.25***	.04	.05	.22***	.28***	-.26***	.30***	.26***	1.00		
10. Avoiding	15.7	3.0	10 to 23	.02	.10*	.10**	-.24***	-.06	.31***	.23***	.10*	-.09	1.00	
11. Fostering reassuring thoughts	11.6	2.8	4 to 19	-.17***	-.15***	.07	.17***	.04	-.01	.38***	-.08	.09*	.45***	1.00

*p<0.05

**p<0.01

***p<0.001

^a^Pairwise correlations with Bonferroni correction

^b^For each subject, a mean was calculated over all beeps. The mean per subject was aggregated over the group to attain a group mean (SD).

^c^PwD = person with dementia

### Predictors of negative affect

The multilevel model estimates of the interaction effects (stress x caregiver characteristic) on negative affect are reported in Table 3.

**Table 3:** Analyses of the daily stress x caregiver characteristics interaction effect on negative affect.

	Negative affect							
	Event-related stress				Activity-related stress			
Caregiver characteristic	B	SE	p	95% CI	B	SE	p	95% CI
Age	-.003	.007	.718	-.016 - .011	.003	.003	.316	-.003 - .010
Gender^a^								
Male	.289	.068	<.001***	.156 - .423	.136	.037	<.001***	.063 - .209
Female	.132	.051	.011*	.031 - .232	.125	.026	<.001***	.074 - .175
Education level: 1–8^a^^b^								
Low: level 1–3	.126	.058	.031**	.012 - .241	.172	.028	<.001***	.117 - .227
Middle: level 4 & 5	.283	.075	<.001***	.136 - .429	.126	.029	<.001***	.069 - .183
High: level 6–8	.200	.103	.053	-.002 - .403	.037	.041	.356	-.042 - .117
Sense of competence	-.005	.009	.564	-.023 - .012	-.011	.004	.002**	-.018 - -.004
Mastery	-.005	.010	.605	-,025 - .014	-.010	.004	.008**	-.017 - -.003
Hours of contact with PwD per week ^c^	.003	.003	.338	-.003 - .010	.001	.002	.632	-.003 - .005
Hours of care for PwD per week^c^	.001	.001	.466	-.001 - .002	.000	.000	.384	-.000 - .001
Coping strategies:								
Active coping	.016	.010	.113	-.004 - .035	-.007	.005	.141	-.016 - .002
Passive coping	.006	.015	.664	-.023 - .035	.008	.007	.260	-.006 - .021
Seeking distraction	-.030	.015	.043*	-.058 - -.001	.001	.007	.864	-.013 - .016
Expressing emotions	-.035	.041	.387	-.116 - .045	-.035	.041	.387	-.116 - .045
Seeking social support	-.028	.014	.046*	-.056 - -.000	.004	.007	.592	-.009 - .017
Avoiding	-.001	.017	.966	-.033 - .032	-.003	.007	.702	-.017 - .012
Fostering reassuring thoughts	-.024	.012	.038*	-.046 - -.001	-.008	.007	.235	-.021 - .005

*p<0.05

**p<0.01

***p<0.001

^a^Effect sizes show the effect of stress on negative affect per stratum of the categorical variable (gender, education level)

^b^Educational level was compressed to three levels: low (primary education, including lower vocational), middle (secondary education, including intermediate vocational), and high (higher education, including higher vocational and bachelor’s, graduate, and doctoral degree).

^c^PwD = person with dementia

With respect to event-related stress, significant interaction effects on negative affect were found with the coping strategies ‘seeking distraction’, ‘seeking social support’, and ‘fostering reassuring thoughts’, indicating that these coping strategies modified the caregivers’ emotional reaction to stress caused by daily events. Caregivers who scored high on these coping strategies experienced less negative affect in reaction to stressful daily events. No significant interaction effects were found with age, sense of competence, mastery, weekly hours of contact with and care for the PwD, and the remaining coping strategies. In addition, post-hoc analyses showed non-significant results for the overall effect of gender (χ^2^ (1) = 3.43, *p* = .064) and educational level (χ^2^ (2) = 2.73, *p* = .255).

With regard to activity-related stress, significant interaction effects on negative affect were found with education level, sense of competence, and mastery. A higher level of education, more sense of competence, and higher levels of mastery lowered caregivers’ emotional reactivity to momentary feelings of stress caused by minor disturbances in daily life. A post-hoc analysis yielded a significant overall effect of education level (χ^2^ (2) = 7.48, *p* = .024). The difference in stress reactivity was present between the low and highly educated caregivers (*B* = -.14, *SE* = .05, *p* = .006). No significant differences were found between the other levels of education (middle versus high: χ^2^ (1) = 3.18, *p* = .075; low versus middle: χ^2^ (1) = 1.29, *p* = .255). Moreover, no significant interaction effects were found with age, hours of contact with and care for the PwD, and coping strategies. In addition, the overall effect of gender was non-significant (χ^2^ (1) = .06, *p* = .799).

Further examination of the relationship between the potential confounding variables (i.e. disease severity and duration, and neuropsychiatric symptoms) and emotional reactivity revealed that only disease duration significantly moderated the association between daily stress and negative affect (*B* = .015, *SE* = .01, *p* = .008). Caregivers who cared for a person with dementia with a longer disease duration experienced more negative affect in reaction to stressful daily events. Adding disease duration as a confounder in the analyses did not affect the results. The addition of neuropsychiatric symptoms, however, led to an increased moderating effect of sense of competence, education, and the coping strategy ‘seeking distraction’ on the association between daily stress and negative affect.

**Fig 1:**
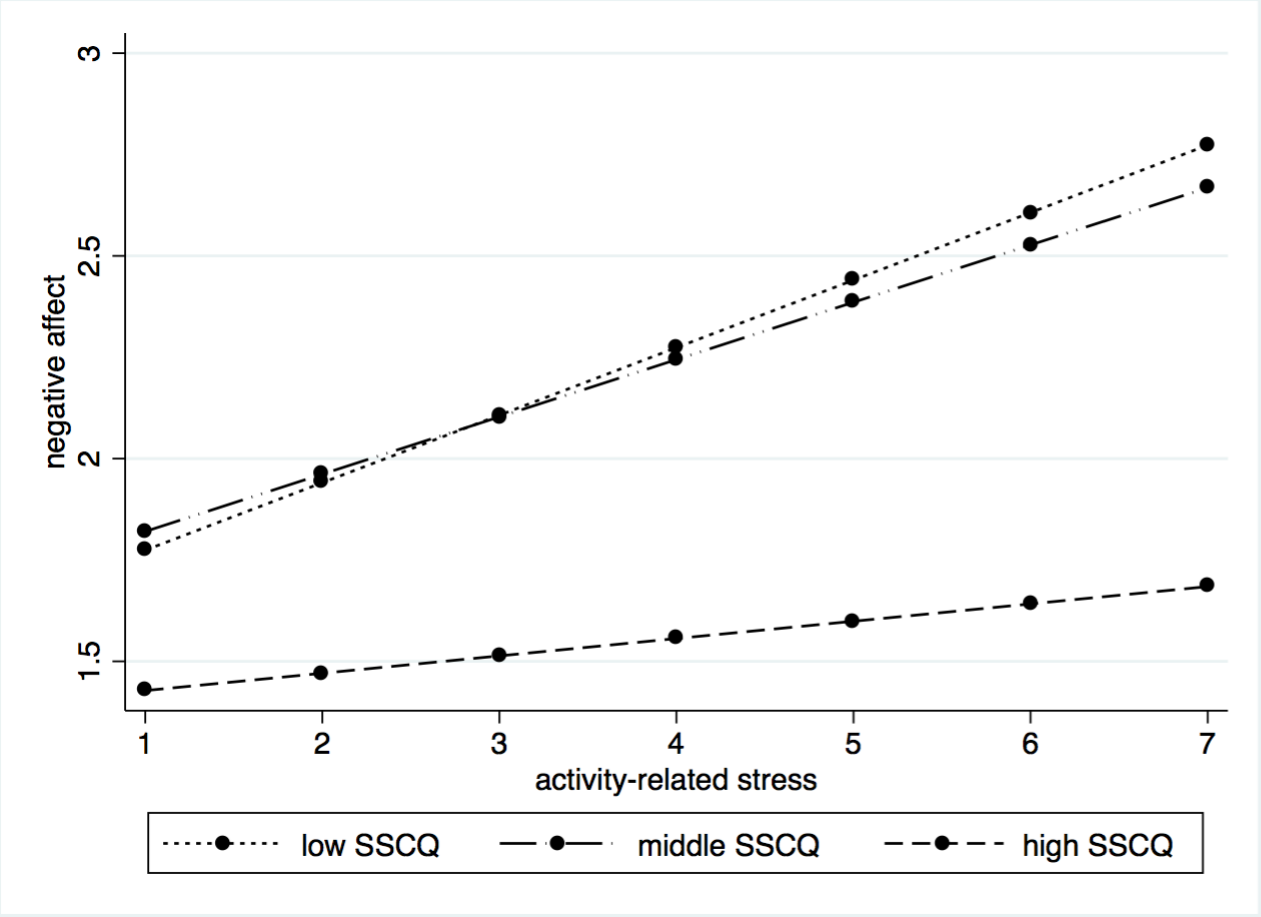
Stratified data illustrating the significant interaction between sense of competence and activity-related stress on negative affect.

Stratified analyses were conducted to further clarify the association between stress and negative affect in relation to sense of competence, mastery, and the coping subscales ‘seeking distraction’, ‘seeking social support’, and ‘fostering reassuring thoughts’. Its results are presented in Table 4. Overall, caregivers with the highest scores (third tertile) on sense of competence, mastery, and the coping strategy ‘seeking social support’ showed a weaker emotional reaction to stress, with smaller increases in negative affect than the caregivers who had middle (second tertile) or low average scores (first tertile). As an example, a graph is included to illustrate the stratified data for sense of competence in more detail (Fig 1).

**Table 4:** Stratified analyses for significant daily stress x caregiver characteristics interaction effects on negative affect^a^.

	Negative affect							
	Event-related stress				Activity-related stress			
Caregiver characteristic	B	SE	p	95% CI	B	SE	p	95% CI
Sense of competence								
Low vs. middle					-.025	.041	.539	-.106 - .055
Low vs. high					-.124	.048	.009**	-.217 - -.031
Middle vs. high					-.099	.046	.033*	-.189 - -.008
Mastery								
Low vs. middle					-.018	.040	.648	-.096 - .060
Low vs. high					-.125	.048	.009**	-.218 - -.031
Middle vs. high					-.106	.049	.031*	-.203 - -.010
Seeking distraction								
Low vs. middle	-.056	.086	.510	-.224 - .111				
Low vs. high	-.204	.118	.085	-.436 - .028				
Middle vs. high	-.148	.122	.224	-.386 - .091				
Seeking social support								
Low vs. middle	.041	.075	.586	-.106 - .188				
Low vs. high	-.262	.101	.009**	-.459 - -.064				
Middle vs. high	-.303	.095	.001**	-.489 - -.116				
Fostering reassuring thoughts								
Low vs. middle	-.081	.093	.385	-.263 - .102				
Low vs. high	-.159	.104	.127	-.363 - .045				
Middle vs. high	-.078	.102	.443	-.278 - .122				

*p<0.05

**p<0.01

^a^Participants were classified into tertiles (low, middle, high) according to their score on the concerning caregiver characteristic. For each caregiver characteristic, emotional reactivity to daily life stress was analyzed in three groups separately according to the following model: negative affect = β0 + β1 stress + residual.

## Discussion

### Main findings

In this study we examined which caregiver characteristics moderate the association between daily stress and negative affect in spousal caregivers of PwD. As suggested in several existing stress models, personal characteristics (e.g. vulnerabilities and resources) may influence the direction of the stress process and play an exacerbating or buffering role in caregivers’ emotional reactivity to daily life stress [6, 8]. In line with previous research by Koerner et al. [46], we found that person-level characteristics can affect the intensity of caregivers’ emotional reactivity to daily life stress. Results showed that caregivers who more frequently used the coping strategies ‘seeking distraction’, ‘seeking social support’, and ‘fostering reassuring thoughts’ reported less emotional reactivity to stressful daily events. In general, coping strategies can be divided into problem-focused (directed at actively altering or managing a problem) and emotion-focused strategies (directed at regulating emotional responses to a problem) [47]. Each form of coping is considered effective under different circumstances. However, problem-focused coping has been found to be conducive to psychological well-being when the stressor is perceived as changeable, whereas emotion-focused coping is more adaptive when the stressor is seen as uncontrollable [48]. It seems plausible that caregivers use both types of coping in response to stressors they encounter in daily life. Our results showed that primarily emotion-focused strategies reduced caregivers’ emotional reactivity when facing stressful daily events. Stressful situations that occur within the presence of the PwD may be related to problem behavior of the PwD, which can be appraised by caregivers as difficult to manage and control [17]. Emotion-focused strategies might, therefore, be more adaptive in these circumstances. Previous studies [46, 49] already demonstrated that emotion-focused strategies could play a buffering role in emotional reactivity to daily life stress. A study on associations between daily coping and end-of-day mood demonstrated that negative affect decreased when distraction and acceptance of the problem were used as coping strategies during times of stress [49]. Furthermore, a study investigating daily stress reactivity among caregivers for elder relatives found that caregivers who reported higher levels of available social support were less reactive to daily fluctuations in care recipient problem behavior [46]. Given that caregivers are at increased risk of becoming socially isolated and often lack social support, an important target in caregiver support interventions could be to stimulate caregivers in seeking social support [50]. In line with our finding that social support appears to be an important resource in buffering against emotional reactivity to daily life stress, social support has been found to enhance caregivers’ feelings of self-worth and self-esteem and to aid in resolving problems or losses [51].

Another finding of our study was that caregivers with a higher education level, more sense of competence, and higher levels of mastery appeared to be less prone to experiencing negative affect when they encountered minor disturbances in daily life. These caregiver characteristics especially seem to play a buffering role when dealing with momentary stressors that continually occur in the flow of daily life. Higher educated caregivers tend to use more effective care management strategies, which may explain their reduced emotional reactivity towards momentary stressors [4]. Caregivers’ sense of competence and mastery have been considered to influence the appraisal of stressful situations and the way in which caregivers cope with distress [38]. A study by Roepke et al. [52] found that caregivers with higher levels of mastery experienced less physical reactivity towards acute psychological stressors, suggesting that mastery also might serve as a resource to reduce emotional reactivity to daily life stress.

In this study caregiver age, gender, and care intensity (i.e. weekly hours of contact with and care for the PwD) did not impact caregivers’ emotional reactivity to daily life stress. Contrary to these findings, a study by Koerner et al. [16] using a daily diary design showed that caregivers experienced an increased level of depressive symptoms and feelings of burden on days when they were involved in more caregiving tasks. Moreover, Koerner et al. [16] proved that female caregivers appeared to be more susceptible to fluctuation in their emotional well-being in the face of daily changes and events in their caregiving role. In addition, Mroczek et al. [53] found a stronger association between daily life stress and negative affect for older as compared to younger adults. Evidently, more studies are needed to further investigate the role of caregiver vulnerabilities (i.e. hard-wired characteristics) in emotional reactivity to daily life stress.

Overall, our results show that primarily caregiver resources (i.e. dynamic characteristics, such as sense of competence, mastery, and coping strategies) can affect emotional reactivity to daily life stress. Differences in emotional reactivity to daily life stress among caregivers might be due to the fact that dynamic caregiver characteristics have the capacity to influence the direction of the stress process and to blunt its impact on caregivers’ negative affect state.

### Implications

Very few studies have observed the caregiving experience from a daily perspective. As a consequence, little is known about emotional reactivity to daily life stress among caregivers of PwD and individual characteristics that could play a buffering or exacerbating role. This gap in the literature is remarkable since understanding emotional reactivity to daily life stress among caregivers in everyday life may be crucial to predict their short-term and long- term mental and physical health [46]. Future studies measuring the day-to-day fluctuations in care-related stressors are, therefore, needed.

Our findings also have important implications for clinical practice. An essential element in successful caregiver support interventions is the focus on personal characteristics and resources [30]. Results of our stratified analyses proved that especially caregivers with high-level resources are less responsive to stress. Intervention programs aimed at reinforcement of caregiver resources, i.e. enhancement of their sense of competence, mastery and coping strategies, could help to reduce caregivers’ emotional reactivity to daily life stress. ESM may be a useful tool to create interventions that are more person-tailored and that provide a more dynamic view on caregiver functioning. Recently, we developed an ESM-based intervention in which caregivers of PwD collect momentary data in their daily lives and receive personalized ESM-derived feedback to increase their sense of competence and mastery in dealing with the daily challenges of dementia [54]. A comparable ESM intervention has proven to be effective in increasing self-awareness and reducing depressive symptoms in persons with depression [55].

### Limitations

The results of this study should be viewed in the light of certain limitations. First, our study sample consisted primarily of caregivers of people with mild stages of dementia. Caregivers reported relatively low levels of negative affect and stress, which might be specific to caregivers who are not yet exposed to high care demands. Moreover, our study sample was highly representative of a memory clinic population, which more often includes relatively young caregivers, who are more pro-active in seeking support [56]. Therefore, the generalizability of the results to a more heterogeneous caregiver population remains unknown. Second, in this study we examined caregivers’ emotional reactivity towards daily stressors in general rather than towards specific care-related stressors. Zooming in to our data, we found that in 71.0% of the reported stressful events the PwD was present. A sensitivity-analysis including only these stressful situations that occurred in the presence of the PwD yielded comparable results with respect to sense of competence, mastery, and seeking social support (results available on request). Finally, emotional reactivity to daily life stress has been defined in terms of emotional reaction to subjective stress. The cross-sectional nature of the data makes it impossible to establish causal relationships. The reverse may also be true in that a worse mood impacts the subjective appraisal of daily stressors. Either explanation, however, has clinical relevance.

## Conclusions

In this study an innovative approach was used to examine caregivers’ stress experiences in the flow of daily life. The results provide evidence that empowerment of caregiver resources, such as sense of competence, mastery, and coping strategies, may help to reduce emotional reactivity to daily life stress among caregivers and could be an important target in caregiver interventions.
